# Dependence of Thermal Comfort of Diving Suit on Neoprene Properties and Diving Depth

**DOI:** 10.3390/polym17212820

**Published:** 2025-10-23

**Authors:** Vesna Marija Potočić Matković, Ivana Salopek Čubrić, Alenka Pavko Čuden

**Affiliations:** 1 Department of Textile Design and Management, University of Zagreb Faculty of Textile Technology, 10000 Zagreb, Croatia; ivana.salopek@ttf.unizg.hr; 2 Faculty of Natural Sciences and Engineering, University of Ljubljana, 1000 Ljubljana, Slovenia; alenka.cuden@ntf.uni-lj.si

**Keywords:** neoprene, diving safety, thermal resistance, compressive stress, compressive displacement, hydrostatic pressure, wetsuit performance, thermal insulation

## Abstract

Neoprene wetsuits experience significant thermal resistance degradation under hydro-static pressure, compromising diver safety and thermal comfort. Despite this known limitation, quantitative predictive models correlating material properties with thermal performance under diving conditions remain underdeveloped. This study quantified thermal resistance changes in commercial neoprene under simulated diving pressure (50, 100, 150, and 200 kPa, equivalent up to a 20 m depth) and developed predictive models for thermal performance degradation. A total of 33 commercially available neoprene sheets representing 11 types in nominal thicknesses of 3, 5, and 7 mm were systematically analyzed. Mass per unit area, thickness, and thermal resistance (R_ct_) were measured under ambient conditions, as was compressive displacement under 50, 100, 150, and 200 kPa compressive loads. Multiple regression analysis established relationships between material properties and thermal performance. Under 200 kPa compression, neoprene samples exhibited compressive displacement ranging from 52.8% to 72.9% (mean: 64.3%). Strong correlations were observed between thermal resistance and thickness (r = 0.9198) and mass per unit area (r = 0.89388). The developed multiple regression model accurately predicted thermal resistance under compression. The 200 kPa pressure-induced thermal resistance reduction ranged from 19.3% to 53.2%, with an average decrease of 40.9%. Even at a pressure of 50 kPa, which corresponds to a diving depth of only 5 m, the thermal resistance of neoprene will be reduced by 21.5% on average. Commercial neoprene demonstrates substantial and predictable thermal performance degradation under diving pressure. The established correlations and predictive models enable evidence-based wetsuit selection and diving safety assessment. These findings highlight the critical need for pressure-resistant thermal insulation technologies and updated diving safety protocols accounting for depth-dependent thermal protection degradation.

## 1. Introduction

Neoprene is a flexible, closed-cell synthetic rubber widely used in the manufacturing of wetsuits due to its excellent thermal insulation, buoyancy, and mechanical resilience. The thermal insulation properties of neoprene stem from its microcellular structure, which traps inert gas (typically nitrogen) in sealed pockets, significantly reducing heat transfer by conduction and convection. Flexibility allows neoprene to conform closely to the body’s shape. It enhances comfort and minimizes water flow between the suit and the skin, which further aids in thermal insulation. One of neoprene’s standout features is its waterproof quality. The closed-cell structure of neoprene stops water from entering, keeping the material dry and maintaining its insulating properties. The fabric ensures water does not soak in, helping to retain warmth even in cold-water environments. Neoprene is a tear-resistant material, able to withstand wear and tear in even the most demanding environments. Its shock absorption capabilities make it suitable for products like protective goods. Neoprene’s resistance to both physical damage and environmental conditions like UV exposure makes it a long-lasting material, perfect for outdoor use [1,2,3,4,5].

However, a known limitation of neoprene is its compressibility under hydrostatic pressure, a factor especially relevant during diving. When a diver submerges, the gas cells compress with increasing depth, which reduces the porosity of the insulation and changes the size and shape of the gas cells. This reduces the thickness and increases the thermal conductivity. The combined effects of the decrease in thickness and increase in thermal conductivity lead to a large reduction in the thermal resistance as a function of depth [6]. Reduced insulation means that divers or users relying on neoprene for warmth could experience much colder conditions than expected, which can affect safety, comfort, and performance underwater. Understanding this loss helps manufacturers improve neoprene products and helps users choose the right thickness and density for their specific needs.

Multiple studies have examined the thermal protection properties and testing methods for diving suits [7,8,9,10,11,12,13,14], but its quantitative prediction for specific commercial materials remains a challenge for manufacturers and end-users. The complex microcellular structure of neoprene, which dictates its initial insulating efficiency, also governs its compressibility. Factors such as cell gas composition, polymer density, and the integrity of the closed-cell foam all influence how the material behaves under hydrostatic load [15,16]. While fundamental models exist to predict the thermal conductivity of foam materials under pressure [15], their application requires detailed knowledge of constituent properties that may not be readily available for commercial, fabric-laminated neoprene sheets.

Recent research has focused on improving material performance and better quantifying the relationship between measurable neoprene properties and thermal behavior under load. Crotti et al. [1] demonstrated the use of infrared sensors and thermodynamic methods to evaluate wetsuit insulation, confirming that material thickness is the primary determinant of thermal resistance. Similarly, Kim and Kim [9] explored the role of sealing technology and foam integrity in preserving heat retention, particularly in cold-water immersion.

Fit and contact with the body have been acknowledged as modifiers of insulation efficiency [2], but for engineering design, standardized test methods like ISO 11092 remain essential for determining intrinsic material properties, such as thermal resistance (R_ct_) and water vapor resistance (R_ct_), under controlled laboratory conditions.

Bardy, Mollendorf, and Pendergast present a correlation that accurately predicts the thermal conductivity of foam neoprene under a range of elevated ambient pressures. This model relies on the known thermal conductivities of the material’s constituents (air and rubber) plus one reference measurement at a baseline pressure. This confirms that the decrease in insulating ability under pressure is a direct result of cell gas compression [15].

Brown et al. [16] investigated the reduction in thermal protection with increasing diving depth. They developed a testing apparatus that enabled the measurement of the material’s thermal insulance in relation to ambient pressure. The results demonstrated that the thermal insulance of 8 mm neoprene decreased by approximately 52% be-tween the surface and a depth of 39 m.

Thicker diving suits provide enhanced thermal protection but tend to compromise flexibility, restrict the range of motion, increase diver fatigue, and experience reduced insulation performance as depth and ambient pressure increase. In contrast, composite materials offer superior thermal protection compared to traditional bubbled neoprene and maintain their insulating properties at greater depths. However, they are less flexible than neoprene and pose challenges in tailoring. In their preliminary study, based on field research, Demers, Martin, and Kartalov [17] developed and tested a segmented diving suit combining neoprene and composite materials, which provides thermal protection that remains largely independent of depth.

Kelly et al. [18] evaluated the thermal protective capability of closed-cell diving suits during cold-water exposure (1.8–2.0 °C) at depths of 9 m, 15 m, and 23 m. Their findings indicated that closed-cell neoprene wetsuits provide adequate protection against hypothermia and non-frostbite cold injury in cold-water dives, with diving depth and duration being critical factors. Variations in core and mean skin temperature were primarily influenced by dive duration rather than depth.

Potočić Matković and Salopek Čubrić [19,20] further emphasized that neoprene’s thickness and mass per unit area can be used to construct reliable predictive models for estimating thermal resistance following compressive deformation. Their findings support the development of regression-based approaches to forecast neoprene insulation performance in underwater environments, facilitating improved design of wetsuits and other aquatic thermal protection systems.

While several studies have characterized neoprene’s compression and thermal degradation under pressure [1,7,14,15], significant gaps remain in the practical application of this knowledge. Existing research has primarily focused on either theoretical models of thermal conductivity [15] or limited experimental datasets [1]. What is critically missing is the following:

- A comprehensive analysis across multiple commercial neoprene types with varying densities, thicknesses, and lining configurations.
- Practical quantification of thermal performance at standardized recreational diving depths (up to 20 m) across diverse commercial products.
- Empirical, property-based models that can bridge the divide between theoretical physics and practical engineering design in the diving industry.

Recent advances in pressure-dependent thermal testing [21] and development of compression-resistant materials [22,23] highlight the urgent need for comprehensive characterization and predictive modeling frameworks. This study addresses these limitations by providing the first multi-type, multi-thickness analysis of commercial neoprene thermal performance under standardized diving pressure, establishing practical predictive models that directly inform diving safety protocols and wetsuit design optimization.

This study aims to address a gap in the practical application of existing theoretical models by providing a robust, empirically derived predictive tool based on readily measurable material properties. While previous studies [15,19,20] have established the theoretical relationship between compression and thermal performance, there is a need for a comprehensive dataset and simple regression models that can directly aid wetsuit designers and material selectors. Therefore, this study analyzes 33 commercially available neoprene samples to achieve the following:

- Quantitatively measure the reduction in thermal resistance (R_ct_) under a compressive load of 50, 100, 150, and 200 kPa, simulating a diving depth of up to 20 m.
- Establish precise correlation coefficients between thermal resistance, thickness, and mass per unit area.
- Develop and validate multiple regression models that can reliably predict the in situ thermal resistance of neoprene after depth-induced compression, using only its initial thickness and mass.

The findings aim to contribute toward a better understanding of material behavior in aquatic thermal protection and support future body-mapped wetsuit designs, material selection, and insulation forecasting for diving equipment.

## 2. Materials and Methods

The neoprene sheets used in this experiment were produced by four well known manufacturers of quality brands (designation M1, M2, M3, M4) in eleven different types (no lining (NO), one-sided lining (OS) and both-sided linings (BS)), with every type in three nominal thickness (3 mm, 5 mm, and 7 mm). These are commonly available thicknesses, but since neoprene sheets are typically manufactured by laminating neoprene with knitted fabrics as lining, the real thickness can be slightly higher than nominal. Altogether, 33 different commercially available neoprene sheets were prepared for the analysis to cover a wide interval of the tested characteristics (Table 1).

The thickness of the neoprene sheets was measured as the vertical distance between the reference plate on which the specimen was placed and the parallel circular plate covering the specimen under pressure, as described in ISO 5084 [24,25]. A DM-2000 thickness gauge (Wolf, Freiberg, Germany) was used for the test. During the test, a pressure of 1 kPa was applied to a sample area of 20 cm^2^. A total of 10 measurements were made at different locations on the specimen. The average thickness result was given as the mean value of 10 measurements.

The mass per unit area of the neoprene sheets was measured using a Kern ALJ 220-4 analytic scale (Kern, Balingen-Frommern, Germany) and following the procedure described in international standard ISO 3801 [26].

A sweating-guarded hotplate (Thermetrics, Seattle, WA, USA), a device that simulates the processes of heat and moisture transfer between the human body and environment, was used to measure the thermal resistance of the neoprene sheets. The testing was conducted at a temperature of 20 °C, with a relative humidity of 65 ± 1% and an air flow velocity of 1 m s^−1^. The resistance of the neoprene sheets was calculated using the following expression [27]:

(1) $$R_{ct}=\frac{\left(T_{s}-T_{a}\right)}{\frac{H}{A}}-R_{ct0}$$

where R_ct_—dry resistance of sample only (m^2^ KW^−1^), T_s_—hotplate surface temperature (°C), T_a_—ambient temperature (°C), H/A—zone heat flux (W m^−2^), and R_ct0_—bare plate dry resistance (m^2^ KW^−1^).

The hydrostatic pressure, P, of sea water at depth depends on the density of the sea water, ρ = 1023.6 kg/m^3^, the acceleration of gravity, g = 9.8 m/s^2^, and the depth of the water column, h, that is, P = ρgh, or 200.75 kPa at a depth of 20 m (or 195.5 kPa with freshwater density). Air pressure was not added, as the result was intended to be relative to the surface pressure. The first neoprene thickness was measured at surface pressure and the second at an added pressure of 200 kPa. Compressive stress of 200 kPa and compressive displacement were measured using an Instron 5567 instrument (Illinois Tool Works Inc., Glenview, IL, USA). Each neoprene sample was measured 5 times, and then the average value was calculated.

## 3. Results

The neoprene sheets examined exhibited a thickness ranging from 3.13 mm to 8.28 mm, with an average of 5.50 mm, reflecting a diverse range of industrially produced neoprene. Mass per unit area displayed substantial variation as well, with values between 687.29 g/m^2^ and 1978.56 g/m^2^, averaging 1323.33 g/m^2^ (Table 1). Compressive stress of 50 kPa induced compressive displacement from 0.95 mm to 2.97 mm with an average of 1.85 mm. With a compressive stress of 200 kPa, compressive displacement at maximum force ranged from 1.99 mm to 5.29 mm, with an average of 3.52 mm, highlighting the material’s varied ability to deform under pressure (Table 2).

Related to the thermal insulation of the investigated neoprene sheets, the results of thermal resistance (R_ct_) testing suggest a wide spread of insulation levels. The range of thermal resistance for the observed specimens is from 0.036 m^2^ KW^−1^ to 0.206 m^2^ KW^−1^ (Table 1), indicating significantly different overall performance in cold environments. The mean thermal resistance for the observed set equals 0.115 m^2^ KW^−1^. It is evident that specimens M3NOc3 and M3NOa3 show notably low values of thermal resistance (0.036 and 0.052 m^2^ KW^−1^), indicating that these neoprene sheets provide lower thermal insulation and are to be perceived as less favorable for cold environments. Conversely, specimens M3NOb7, M4BS7, and M4OS7 have the highest values of thermal resistance. This labels them as materials with better thermal insulating properties and more appropriate for the clothing needed for a longer stay in a colder environment.

## 4. Discussion

**Figure 1:**
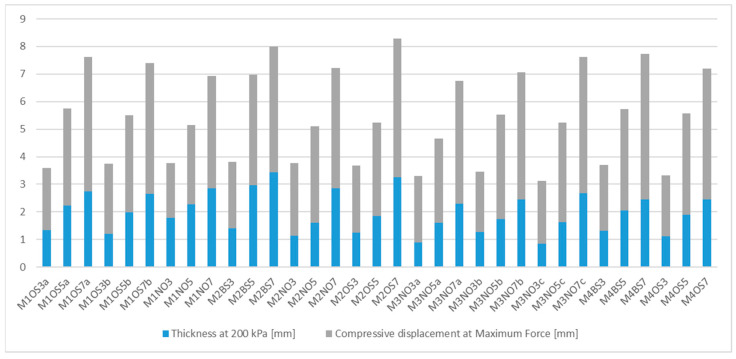
Initial thickness of the neoprene compared to thickness under compressive stress of 200 kPa.

Figure 1 shows the thickness of the neoprene under a pressure of 200 kPa, as well as the compressive displacement. Together, they give the initial thickness of the neoprene. When subjected to 200 kPa of pressure, the neoprene sheets exhibited an average thick-ness reduction of 64.3%. No statistically significant differences in compression were observed either between manufacturers or between samples with one-sided versus two-sided lamination. These findings suggest that the compression response is largely governed by the intrinsic properties of the material rather than production variations.

The compressed thickness is strongly linked to other measurable material characteristics. The thickness at 200 kPa correlates closely with the original thickness at 1 kPa (r = 0.9476), while the compressive displacement is highly correlated with the original thickness (r = 0.9755) and somewhat less with mass per unit area (r = 0.8821) (Table 3). This confirms that the compressed thickness can be reliably estimated from basic properties and reinforces its role as the most relevant predictor for subsequent thermal behavior.

The thermal resistance of neoprene depends not only on the thickness but also on the cell foam structure; in the closed-cell foam structure, gas-filled bubbles are sealed and do not interconnect; also, a lower density (more air/gas, less solid rubber) generally means lower thermal conductivity. Three of the eleven groups of neoprene show these characteristics (M3NOb, M4BS, M4OS), although with the same thickness, a stronger thermal resistance is evident, so these three neoprene groups will be considered separately for statistical reasons (Table 1).

The thermal resistance of neoprene was found to be highly correlated with its thickness (r = 0.9198) and, to a slightly lesser extent, with its mass per unit area (r = 0.89388), as shown in Table 4. This relationship is expressed by the multiple regression equation:

R_ct_ = −0.000561 + 0.0124136 × Thickness + 0.0000201 × Mass per unit area (2)

The statistical evaluation of the model yielded a *p*-value of 1.75126 × 10^−9^ a correlation coefficient (multiple R) between observed and predicted values of 0.923814875, and a coefficient of determination (R^2^) of 0.853433924, indicating that the model is statistically significant.

Based on these results, it is possible to reliably estimate the thermal resistance after compressive displacement by inputting the thickness at 200 kPa into the given formula.

Using the same method, an estimation of thermal resistance was performed for the remaining three neoprene samples of lower mass but higher thermal resistance (high-end neoprene: M3NOb, M4BS, M4OS). The thermal resistance of these neoprenes was found to be even more strongly correlated with thickness (r = 0.98027) and mass per unit area (r = 0.94059), as shown in Table 5. The relationship is described by the following multiple regression equation:

R_ct_ = 0.06196 + 0.01478 × Thickness + 0.00002025 × Mass per unit area (3)

The model yielded a *p*-value of 3.60343 × 10^−5^, a correlation coefficient (multiple R) of 0.98334645 between observed and predicted values, and a coefficient of determination (R^2^) of 0.966970242, indicating high statistical significance.

This enables a relatively reliable estimation of thermal resistance after compressive displacement for these three additional samples as well by entering the thickness at 200 kPa into the given equation.

The high correlation between compressive displacement and original thickness (r = 0.9755) indicates that, for the range of commercial materials tested, the initial thickness is the primary predictor of how much a neoprene sheet will compress at 20 m. This simplifies the forecasting process for designers. The developed regression models offer a significant practical advantage over more complex theoretical approaches [7] by requiring only two easily measurable inputs: initial thickness and mass per unit area. The exceptional performance of the M3NOb, M4BS, and M4OS groups, which required a separate, even more accurate model (R^2^ = 0.967), underscores the impact of advanced foam morphology. These materials likely possess a more optimal cell size distribution and higher closed-cell content, maximizing gas entrapment per unit mass and thus thermal resistance, as suggested by Kim and Kim [5]. This highlights an avenue for future material development: engineering neoprene foams that not only have low density but also a cell structure that resists collapse under pressure.

**Figure 2:**
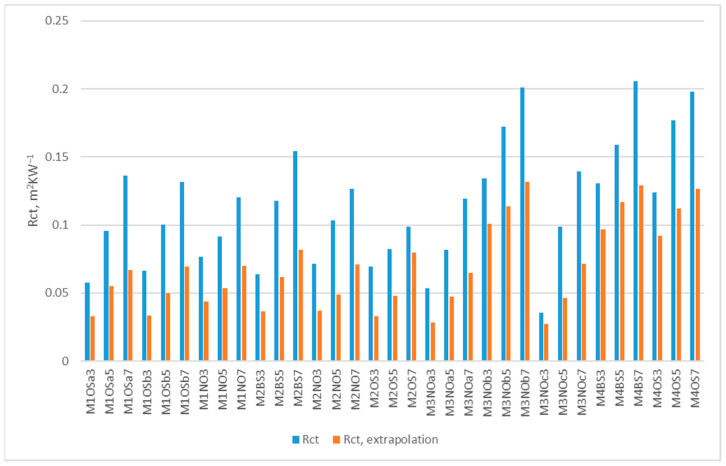
Extrapolated results of thermal resistance of neoprene sheets at a depth of 20 m compared with thermal resistance at the surface.

The results of the extrapolation are presented in Figure 2 for all analyzed neoprene samples. Based on the initial thickness and mass of each neoprene, and by applying the thickness at 200 kPa into the previously defined multiple regression equation—which describes the relationship between thickness, mass, and thermal resistance—the estimated thermal resistance was found to decrease between 19.3% and 53.2%, with an average reduction of 40.9%. This suggests that at a depth of 20 m, neoprene loses approximately 40.9% of its thermal insulating capacity.

**Figure 3:**
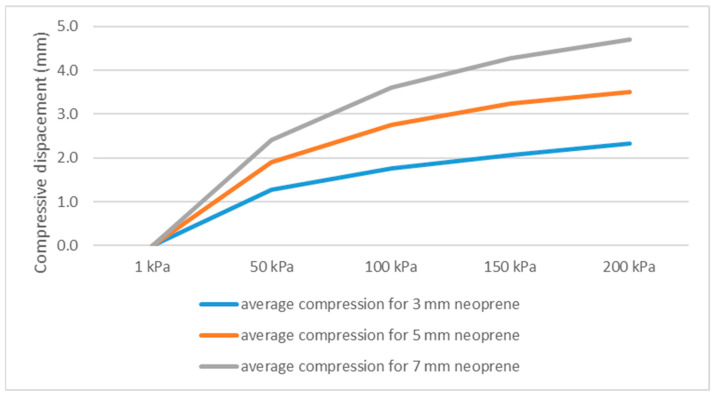
Compressive displacement under different pressures corresponding to different diving depths.

It should be noted that the estimation of thermal resistance would vary at different depths. The compressive displacement was measured at pressures corresponding to different diving depths (5, 10, 15, 20 m), with 200 kPa selected as the upper reference point, equivalent to approximately 20 m depth, which corresponds to the limit for amateur divers. It is important to predict the behavior of neoprene even at shallower depths. The average compression values for neoprene were 1.86 mm at 50 kPa, 2.70 mm at 100 kPa, 3.19 mm at 150 kPa, and 3.52 mm at 200 kPa, respectively (Figure 3). As expected, the thinnest neoprene samples (3 mm) exhibited the lowest average displacement at all pressures, while the thickest (7 mm) showed the highest, consistent with their larger initial volume and greater compressible capacity (Figure 3). The observed nonlinear behavior indicates that most of the structural collapse of gas-filled cells happens at shallower depths, whereas at greater depths, the compression stabilizes. This behavior is particularly important in evaluating wetsuit performance, since the initial rapid compression within the first 5–10 m of immersion substantially reduces the thickness and thereby the thermal insulation capability of the neoprene material (Figure 3).

**Figure 4:**
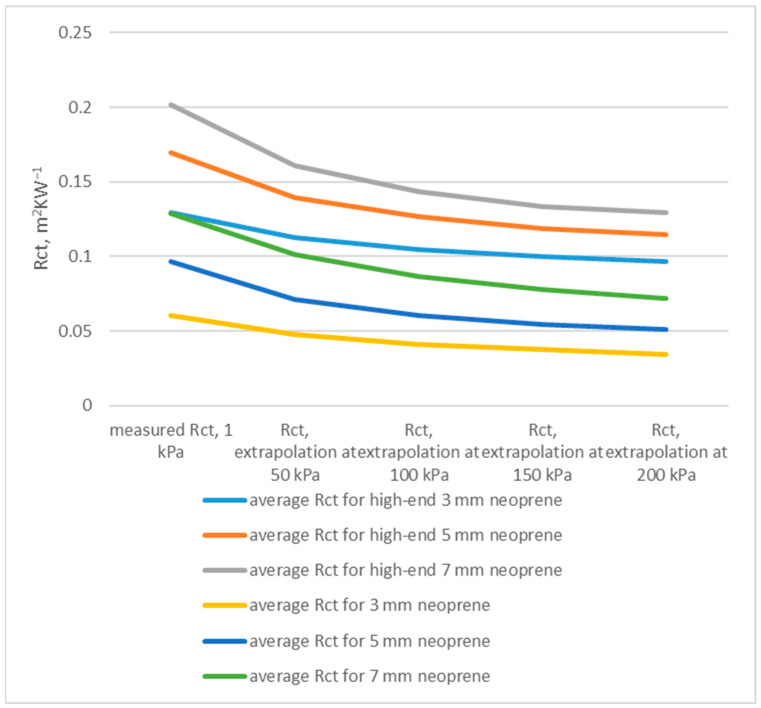
Estimated thermal resistance under different pressures corresponding to different diving depths.

The results of the thermal resistance measurements for both standard and high-end neoprene sheets confirm that compressive displacement has a pronounced effect on the insulation capacity of neoprene, especially within the first few meters of immersion. As the hydrostatic pressure increases with depth, the gas-filled cells within the material collapse, reducing its thickness and the air volume responsible for thermal insulation. This process occurs most rapidly within the first 5 m of descent (≈50 kPa) and then gradually slows as further compression becomes more limited. For high-end neoprene, the average thermal resistance at surface pressure (1 kPa) and at 50 kPa decreased from 0.1295 to 0.1123 m^2^ KW^−1^ (−13.3%) for 3 mm sheets, from 0.1693 to 0.1395 m^2^ KW^−1^ (−17.6%) for 5 mm sheets, and from 0.2015 to 0.1609 m^2^ KW^−1^ (−20.1%) for 7 mm sheets (Figure 4).

In contrast, standard neoprene exhibited a significantly higher loss, from 0.0607 to 0.0479 m^2^ KW^−1^ (−21.0%) for 3 mm, from 0.0964 to 0.0708 m^2^ KW^−1^ (−26.6%) for 5 mm, and from 0.1284 to 0.1009 m^2^ KW^−1^ (−21.4%) for 7 mm sheets (Figure 4). These results indicate that a considerable portion of thermal insulation is lost within the first 5–10 m of immersion, where most compressive displacement takes place. High-end neoprene, owing to its finer cell structure and improved elastic recovery, maintains a higher residual thermal resistance at all pressure levels, retaining up to 5–10% more insulation than standard materials of equivalent thickness.

At greater depths (beyond ~10 m), both materials show a slower decline in R_ct_ values, consistent with the stabilization of compressive deformation as the internal cell structure becomes saturated and less responsive to additional pressure.

It should be noted that these estimations reflect only the properties of the neoprene sheets. In real diving conditions, other factors influence thermal performance: The thin water film trapped between the skin and suit can aid or hinder insulation depending on circulation, while suit fit is critical in preventing flushing. The fit of the wetsuit to the body is another important factor influencing heat retention, which was not accounted for in this study. It would be valuable to conduct further research involving temperature sensors placed between the body and the wetsuit to record the actual skin temperature during diving and compare it with the extrapolated results presented here.

The 40.9% average reduction in thermal resistance at 20 m depth has significant practical implications for dive safety and comfort. For a diver wearing a 5 mm wetsuit with an initial thermal resistance of 0.12 m^2^ KW^−1^, depth-induced compression would reduce this to approximately 0.07 m^2^ KW^−1^, potentially insufficient for extended dives in water below 15 °C. This suggests that current wetsuit selection guidelines, often based solely on surface-level thermal testing, may underestimate thickness requirements for deeper recreational diving.

Furthermore, the wide range of thermal resistance loss across samples indicates that material selection matters significantly. High-performance neoprene, evident in the M3NOb, M4BS, and M4OS groups, offers not only better surface insulation but also superior depth retention, making it more cost-effective for serious divers despite higher initial purchase costs.

## 5. Conclusions

Based on a comprehensive analysis of 33 commercial neoprene samples, this study provides quantitative evidence and predictive tools for assessing thermal performance loss in diving wetsuits. The key findings are as follows:

1. The thermal resistance of neoprene at the surface is highly variable (0.036–0.206 m^2^ KW^−1^) and is predominantly determined by its thickness and mass per unit area.
2. Under a pressure of 200 kPa (simulating 20 m depth), neoprene exhibits substantial compressibility, with an average thickness reduction of 64.3%. This compression is directly correlated with the original thickness of the material.
3. The consequent average loss in thermal resistance is 40.9%, a critical figure for diver safety and comfort planning. The developed multiple regression models enable reliable prediction of this loss based on simple initial material measurements.
4. Neoprene’s thermal resistance decreases rapidly within the first 5–10 m of immersion due to intensive compressive displacement, after which the rate of degradation slows significantly. High-end neoprene materials exhibit improved structural stability and retain up to 10% more thermal insulation compared to standard neoprene, highlighting their advantage for wetsuits intended for prolonged or deeper recreational dives.
5. Multiple regression models successfully predicted thermal resistance from thickness and mass per unit area, with high statistical significance (R^2^ = 0.853 for standard neoprene, R^2^ = 0.967 for high-performance variants, *p* < 0.001). These models enable reliable estimation of depth-dependent thermal performance from surface measurements, supporting evidence-based material selection.

## Figures and Tables

**Table 1:** Measured properties of thickness, mass per unit area, and thermal resistance of neoprene sheets.

Sample	Thickness at 1 kPa, mm	Mass Per Unit Area, gm^−2^	Thermal Resistance, R_ct_
M1OSa3	3.5900	826.61	0.0575
M1OSa5	5.7567	1380.69	0.0955
M1OSa7	7.6233	1668.22	0.1365
M1OSb3	3.7400	945.70	0.0665
M1OSb5	5.5033	1277.17	0.1005
M1OSb7	7.3967	1835.69	0.1315
M1NO3	3.7667	1104.54	0.0765
M1NO5	5.1467	1283.13	0.0915
M1NO7	6.9400	1755.99	0.1205
M2BS3	3.8067	968.12	0.0635
M2BS5	6.9833	1277.00	0.1175
M2BS7	8.0000	1966.54	0.1545
M2NO3	3.7633	1148.23	0.0715
M2NO5	5.1000	1458.62	0.1035
M2NO7	7.2167	1808.25	0.1265
M2OS3	3.6733	895.70	0.0695
M2OS5	5.2367	1277.00	0.0825
M2OS7	8.2833	1978.56	0.0985
M3NOa3	3.2933	874.61	0.0535
M3NOa5	4.6567	1382.11	0.0815
M3NOa7	6.7433	1820.76	0.1195
M3NOb3	3.4500	988.29	0.1340
M3NOb5	5.5167	1288.65	0.1720
M3NOb7	7.0667	1659.18	0.2010
M3NOc3	3.1267	858.12	0.0355
M3NOc5	5.2467	1323.01	0.0985
M3NOc7	7.6267	1933.70	0.1395
M4BS3	3.6967	759.59	0.1305
M4BS5	5.7167	1202.27	0.1590
M4BS7	7.7367	1538.36	0.2055
M4OS3	3.3167	687.29	0.1240
M4OS5	5.5733	1093.46	0.1770
M4OS7	7.1967	1404.61	0.1980

Designation M1, M2, M3, and M4 for 4 different manufacturers, no lining on neoprene (NO), one-sided lining (OS), both-sided linings (BS), nominal thickness (3 mm, 5 mm, and 7 mm).

**Table 2:** Measured properties compressive displacement under 50, 100, 150, and 200 kPa.

Sample	Compressive Displacement at 50 kPa, mm	Compressive Displacement at 100 kPa, mm	Compressive Displacement at 150 kPa, mm	Compressive Displacement at 200 kPa, mm
M1OSa3	0.9480	1.4424	1.7454	2.2409
M1OSa5	1.8563	2.6931	3.1880	3.5361
M1OSa7	2.6134	3.8141	4.5001	4.8921
M1OSb3	1.2812	1.8560	2.1887	2.5285
M1OSb5	1.8770	2.7348	3.2186	3.5175
M1OSb7	2.5327	3.7126	4.3689	4.7489
M1NO3	1.0289	1.5539	1.8567	1.9871
M1NO5	1.3917	2.1887	2.6430	2.8679
M1NO7	1.8575	2.9068	3.5125	4.0899
M2BS3	1.3424	1.8770	2.1803	2.3915
M2BS5	2.2996	3.2784	3.8224	4.0158
M2BS7	1.8867	3.0666	3.7524	4.5651
M2NO3	1.4429	2.0482	2.3912	2.6147
M2NO5	1.9373	2.7539	3.2173	3.4899
M2NO7	2.2498	3.4194	4.0652	4.3669
M2OS3	1.1504	1.7260	2.0488	2.4266
M2OS5	1.8154	2.6728	3.1571	3.3950
M2OS7	2.3291	3.6905	4.4471	5.0344
M3NOa3	1.3931	1.9274	2.2296	2.4007
M3NOa5	1.7240	2.4711	2.8945	3.0578
M3NOa7	2.3106	3.4406	4.0765	4.4477
M3NOb3	0.9581	1.4729	1.7758	2.1753
M3NOb5	2.1198	3.0483	3.5628	3.7701
M3NOb7	2.5008	3.6812	4.3166	4.6160
M3NOc3	1.2918	1.7765	2.0594	2.2801
M3NOc5	1.9673	2.8044	3.2690	3.6280
M3NOc7	2.5720	3.8026	4.4792	4.9447
M4BS3	1.4129	1.9376	2.2498	2.3798
M4BS5	1.9768	2.8548	3.3490	3.6564
M4BS7	2.9671	4.2378	4.9239	5.2902
M4OS3	1.2209	1.7256	2.0181	2.2109
M4OS5	1.8857	2.7433	3.2482	3.6741
M4OS7	2.7534	3.9029	4.5375	4.7555

Designation M1, M2, M3, and M4 for 4 different manufacturers, no lining on neoprene (NO), one-sided lining (OS), both-sided linings (BS), nominal thickness (3 mm, 5 mm, and 7 mm).

**Table 3:** Correlation between the compressive displacement, mass per unit area, and thickness of neoprene.

	Compressive Displacement at Maximum Force, mm	Thickness at 1 kPa, mm	Thickness at 200 kPa, mm	Mass Per Unit Area, gm^−2^
Compressive displacement at maximum force, mm	1			
Thickness at 1 kPa, mm	0.97550	1		
Thickness at 200 kPa, mm	0.85406	0.94757	1	
Mass per unit area, gm^−2^	0.88211	0.91189	0.87490	1

**Table 4:** Correlation between the thermal resistance, thickness, and mass per unit area of neoprene sheets.

	Thickness at 1 kPa, mm	Mass Per Unit Area, gm^−2^	Thermal Resistance, m^2^ KW^−1^
Thickness at 1 kPa, mm	1.00000		
Mass per unit area, gm^−2^	0.93988	1.00000	
Thermal resistance, m^2^ KW^−1^	0.91980	0.89388	1

**Table 5:** Correlation between the thermal resistance, thickness, and mass per unit area of M3NOb, M4BS, and M4OS neoprene sheets.

	Thickness at 1 kPa, mm	Mass Per Unit Area, gm^−2^	Thermal Resistance, m^2^ KW^−1^
Thickness at 1 kPa, mm	1.00000		
mass per unit area, gm^−2^	0.93047	1.00000	
Thermal resistance, m^2^ KW^−1^	0.98027	0.94059	1

## Data Availability

The raw data supporting the conclusions of this article will be made available by the authors on request.
